# Insecticide resistance status in the whitefly, *Bemisia tabaci* genetic groups Asia-I, Asia-II-1 and Asia-II-7 on the Indian subcontinent

**DOI:** 10.1038/srep40634

**Published:** 2017-01-18

**Authors:** N. C. Naveen, Rahul Chaubey, Dinesh Kumar, K. B. Rebijith, Raman Rajagopal, B. Subrahmanyam, S. Subramanian

**Affiliations:** 1 Indian Agricultural Research Institute, Division of Entomology, New Delhi, 110012, India; 2 Banaras Hindu University, Department of Zoology, Varanasi, 221005, India; 3 University of Cambridge, Department of Physiology, Development and Neuroscience, Cambridge, CB2 3EG, United Kingdom; 4 University of Delhi, Department of Zoology, Delhi, 110007, India

## Abstract

The present study is a summary of the current level of the insecticide resistance to selected organophosphates, pyrethroids, and neonicotinoids in seven Indian field populations of *Bemisia tabaci* genetic groups Asia-I, Asia-II-1, and Asia-II-7. Susceptibility of these populations was varied with Asia-II-7 being the most susceptible, while Asia-I and Asia-II-1 populations were showing significant resistance to these insecticides. The variability of the LC_50_ values was 7x for imidacloprid and thiamethoxam, 5x for monocrotophos and 3x for cypermethrin among the Asia-I, while, they were 7x for cypermethrin, 6x for deltamethrin and 5x for imidacloprid within the Asia-II-1 populations. When compared with the most susceptible, PUSA population (Asia-II-7), a substantial increase in resistant ratios was observed in both the populations of Asia-I and Asia-II-1. Comparative analysis during 2010–13 revealed a decline in susceptibility in Asia-I and Asia-II-1 populations of *B. tabaci* to the tested organophosphate, pyrethroid, and neonicotinoid insecticides. Evidence of potential control failure was detected using probit analysis estimates for cypermethrin, deltamethrin, monocrotophos and imidacloprid. Our results update resistance status of *B. tabaci* in India. The implications of insecticide resistance management of *B. tabaci* on Indian subcontinent are discussed.

The whitefly, *Bemisia tabaci* Gennadius (Hemiptera: Aleyrodidae), is one of the world’s top 100 invasive organisms^1^. It is causing severe economic damage in over 60 crop plants as a phloem sap sucking pest or as a vector of viral diseases^2^. Wider host adaptability, cryptic species status, and virus transmission capabilities have rendered the management of this pest very difficult^1^. *B. tabaci* has tremendous potential to develop resistance to insecticides. To date, *B. tabaci* has shown resistance to more than 40 active ingredients of insecticides^3^.

Historically, cotton and vegetables have accounted for more than 50 percent of insecticide usage in India^4^. With the wider adoption of *Bt* cotton technology in India during 2002, the insecticide usage on cotton for controlling bollworms had started declining^5^. However, there has been a surge in demand for insecticides on cotton since 2006. As per one estimate, the insecticide usage on cotton in India has increased from 2374 MT in 2006 to 6372 MT in 2011, on account of increase in area under sucking pest susceptible *Bt* cotton hybrids, resurgence of sucking pests and due to progressive increase in levels of resistance by sucking pests to insecticides^4^,^6^,^7^.

Insecticides have been the mainstay of controlling *B. tabaci* in diverse agricultural production systems. Organophosphates (OPs) and organochlorine insecticides had been gradually replaced by pyrethroids during the late 70s and 80s^8^. Subsequently, the OPs and pyrethroids have been replaced by neonicotinoids and other compounds of novel chemistry during the late 90s, worldwide^9^. Nevertheless, continued use of these compounds for controlling sucking insects such as *B. tabaci* occurs on the Indian subcontinent^7^,^10^. Several field problems such as poor selection of chemicals and sub-standard application practices exacerbated the control failures of insecticides against *B. tabaci* in India^11^. The repeated use of compounds of same active ingredients and application of excessive doses of insecticides within a given cropping season has led to the development of insecticide resistance against OPs and pyrethroids in *B. tabaci*^12^,^13^.

Resistance to insecticides resulting in loss of efficacy of many older insecticides has placed excessive pressure on novel products^14^. Studies have shown the development of resistance in whiteflies to even compounds of novel chemistry in several countries, including Brazil^15^,^16^, Burkina Faso^17^, China^18^,^19^, Colombia^20^, Cyprus^21^, Egypt^22^, Germany^23^,^24^, Greece^25^, Guatemala^26^, India^12^,^27^, Iran^28^, Israel^29^,^30^,^31^,^32^,^33^, Italy^34^, Malaysia^35^, Nicaragua^36^, Pakistan^37^, Spain^16^, Sudan^36^, Turkey^38^, and USA^39^,^40^,^41^,^42^,^43^,^44^,^45^. India has a long history of resistance to OPs, pyrethroids, and carbamates by bollworms, *Helicoverpa armigera* (Hübner) and whitefly on cotton^12^,^27^,^46^,^47^,^48^. Further, some researchers observed that the preponderance of *B. tabaci* genetic groups in certain geographical regions had principally been driven by insecticide tolerance levels in specific *B. tabaci* genetic groups^30^,^49^,^50^. The dominance of B and Q biotypes over indigenous biotypes of *B. tabaci* especially in China, Israel, North America was largely attributed to their insecticide resistance traits^19^,^31^,^42^,^51^. Extensive information is available on the insecticide resistance status of Mediterranean (MED) and the Middle East-Asia Minor 1 (MEAM 1) genetic groups, known in older literature as the Q and B biotypes, respectively^1^. Although Indian geographical regions display an enormous diversity of *B. tabaci* with the presence of nine out of the 36 genetic groups recorded so far^52^,^53^, only limited literature is available on the insecticide resistance status of Indian contingent of *B. tabaci* species complex^10^,^12^,^27^. The present investigation attempts to take a snapshot view of resistance development in field populations of *B. tabaci* (collected across agro-climatic zones) against OPs, synthetic pyrethroids and neonicotinoids concurrently used for controlling *B. tabaci* in India along with information on their genetic group status. Besides, the changes in susceptibility levels of selected *B. tabaci* field populations against OP, pyrethroid, and neonicotinoid compounds were estimated from 2010 to 2013 for understanding the dynamics of insecticide resistance development in these *B. tabaci* populations.

Insecticide resistance is often manifested as control failures at field level. Recent studies in Brazil and Greece^54^,^55^ explored insecticide resistance of tomato leafminer, *Tuta absoluta* (Meyrick) deploying analytical tools to estimate the potential control failures. This study attempts to predict potential control failures of the commonly used OP, pyrethroid and neonicotinoid compounds in regional, Indian populations of *B. tabaci* using probit analysis of existing populations and comparing them to a susceptible population.

## Results

### Genetic group status of *B. tabaci* populations

**Figure 1:**
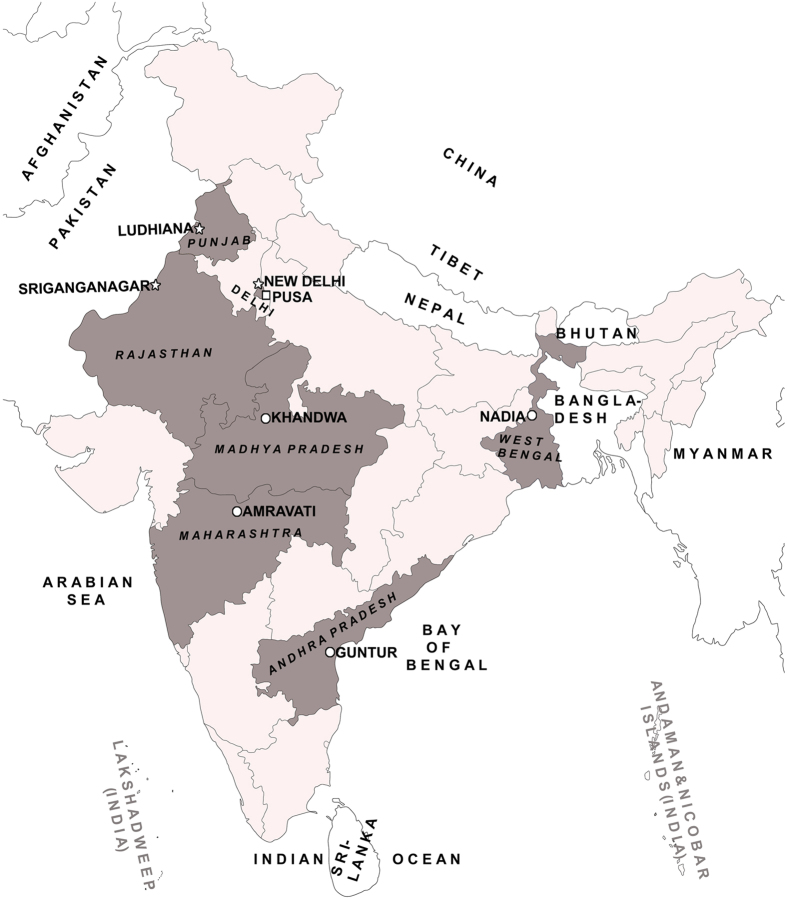
The map shows the survey locations and distributions of *B. tabaci* populations in India. On India map, the states are delimited by thin lines with states in light gray indicate the collection regions. Collection sites are indicated by names and markings; genetic groups of *B. tabaci* are indicated by different symbols: circle-Asia-1, polygon-Asia-II-1, and square-Asia-II-7. The image was acquired from http://d-maps.com/carte.php?num_car=4183&lang=en; the final image was created using the software Adobe Photoshop Version 7.0 (Adobe Systems, San Jose, CA, USA).

The genetic group status and geographical information of all the *B. tabaci* populations used in this study are shown in Table 1 and Fig. 1. The mitochondrial cytochrome oxidase 1 sequence analysis showed that each of the *B. tabaci* populations could be assigned to a single genetic group and it was observed that there was no mixture of different genetic groups in any of the populations. Three *B. tabaci* populations from Ludhiana, Sriganganagar, and New Delhi were assigned to the Asia-II-1 genetic group, while the populations from Amravati, Khandwa, Guntur, and Nadia belonged to the Asia-I genetic group. The *B. tabaci* population collected from the cotton fields of the Indian Agricultural Research Institute, Pusa Campus, New Delhi (designated as PUSA population) was assigned to the Asia-II-7 genetic group. The representative sequences of all populations used in this study were deposited in GenBank under accession numbers KF298445 to KF298451, KP641660, and KU613373.

### Insecticide usage history and cropping details

The details of Knowledge-Attitude-Practice surveys are presented in Table 1. The surveys were conducted in farmers’ fields in the study locations before the start of this investigation to collect primary data on the cropping and insecticide usage pattern of the farmers in these localities. The surveys revealed that the commercial *Bt* cotton hybrid seeds available to the farmers had been pre-treated with imidacloprid 70WS; whitefly, *B. tabaci*, and the leafhopper, *Amrasca biguttula biguttula* (Ishida) were the major sucking pests on cotton in northern and southern India, while the whiteflies were the predominant sucking pests on brinjal in eastern India. The OPs, pyrethroids, and neonicotinoids were predominantly used by the farmers for control of whitefly in cotton (and brinjal in Nadia) in these regions. The number of spray applications was 10–12 in Nadia (Eastern India), 7–10 in Ludhiana and Sriganganagar locations (Northwestern India), 6–8 in Guntur (Southern India) and 4–6 in Amravati and Khandwa (Central India).

### Insecticide bioassays

Insecticide bioassays were conducted in 2013 to generate dose response data for the *B. tabaci* populations (from different geographic locations) against OP, pyrethroid, and neonicotinoid compounds. The results of dose response regressions analyzed by probit analysis are shown in Table 2. The χ^2^ analysis showed that dose responses of all the tested populations fitted the log-dose probit mortality model and the linearity was rejected only for cypermethrin against New Delhi and for Nadia populations (Table 2). Resistance ratios were computed separately for Asia-I and Asia-II-1 populations in comparison to the most susceptible *B. tabaci* population within the respective genetic groups for each insecticide. In the absence of a characterized susceptible strain, we have also computed resistance ratios for the field populations using the PUSA population (Asia-II-7) as the reference check (as it had significantly lower lethal concentration values for all the tested insecticides).

### Pyrethroids

The tested *B. tabaci* populations exhibited the highest slopes in response to the pyrethroids. The slopes of probit response curves ranged from 1.32 to 2.89 for cypermethrin and 1.33 to 4.81 for deltamethrin.

The LC_50_ values for cypermethrin were in the range of 194 to 1362 mg L^−1^ among the Asia-I populations, and 238 to 701 mg L^−1^ among the Asia-II-1 populations. There was upto threefold increase in resistance ratio in Khandwa (Asia-I); four and a sevenfold increase in resistance ratio values respectively in Ludhiana and Sriganganagar (Asia-II-1) in comparison to the most susceptible populations within the respective genetic groups. However, the magnitude of resistance was high in comparison to the PUSA with Sriganganagar and Ludhiana populations recording respectively 136 and 78 fold resistance to cypermethrin, while, the Khandwa population was showing 70 fold resistance to this pyrethroid. The LC_50_ values for deltamethrin ranged from 120 to 760 mg L^−1^ in the Asia-II-1 populations and 128 to 242 mg L^−1^ in the Asia-I populations. Ludhiana and Sriganganagar showed respectively 76 and 71 fold resistance to deltamethrin in comparison to the PUSA (Table 2).

### Organophosphates

Triazophos, monocrotophos, and chlorpyrifos were the tested OP compounds. The slopes of the response lines ranged from 1.50 to 2.86 for triazophos; 1.35 to 1.87 for the monocrotophos and 1.57 to 2.34 for chlorpyrifos. For triazophos, the LC_50_ values ranged from 324 to 525 mg L^−1^ in Asia-II-1 and 445 to 1429 mg L^−1^ in Asia-I populations of *B. tabaci*. A threefold increase in resistance ratio to triazophos was observed in Nadia (Asia-I), while, resistance to triazophos was not significant among the Asia-II-1 populations. The Nadia population was found to be showing 27 fold resistance to triazophos in comparison to the reference PUSA population (Table 2). Analysis of the dose response to monocrotophos showed that the LC_50_ values were ranging from 528 to 2114 mg L^−1^ and 843 to 3934 mg L^−1^ respectively, in the Asia-II-1 and Asia-I populations resulting in fourfold resistance in Ludhiana and fivefold resistance in Nadia in comparison to the susceptible checks within the respective genetic groups. However, the Nadia (Asia-I) and Ludhiana (Asia-II-1) populations recorded significantly higher resistance ratios of 44 and 24 in comparison to the PUSA (Asia-II-7). Among the OP compounds, the chlorpyrifos recorded significantly lower LC_50_ values of 137 to 201 mg L^−1^ and 56 to 309 mg L^−1^ respectively in the Asia-II-1 and Asia-I populations. Comparisons within Asia-I and Asia-II-1 showed a fivefold increase in resistance ratio to chlorpyrifos in Guntur (Asia-I), while no significant increase in resistance ratio was noticed among the Asia-II-1 populations. However, the two Asia-I populations from Guntur and Amravati were showing respectively 25 and 18 fold resistance to chlorpyrifos in comparison to the PUSA population.

### Neonicotinoids

Imidacloprid and thiamethoxam were the tested neonicotinoids. The slopes of the response lines to imidacloprid ranged from 1.37 to 2.23 and 1.52 to 2.11 respectively in Asia-I and Asia-II-1 populations. The LC_50_ values were in the range of 178 to 901 mg L^−1^ and 130 to 956 mg L^−1^ respectively, for the Asia-II-1 and Asia-I populations. Sriganganagar and Nadia were showing respectively fivefold and sevenfold resistance to imidacloprid in comparison to the most susceptible population within the respective genetic groups (Table 2). But, these two populations were found to be showing respectively 18 and 17 fold resistance to imidacloprid in comparison to the PUSA population. For thiamethoxam, the LC_50_ values were ranging from 73 to 194 mg L^−1^ and 23 to 179 mg L^−1^ respectively, for the Asia-II-1 and Asia-I populations resulting in upto sevenfold increase in resistance ratio of Guntur (Asia-I) & Amravati (Asia-I) and twofold increase in resistance ratio of Sriganganagar (Asia-II-1) in comparison to the susceptible checks within the respective genetic groups. However, in comparison to PUSA, Sriganganagar, Amravati and Guntur populations showed about a sevenfold increase in resistance ratios to thiamethoxam (Table 2).

### Pairwise correlation analysis of LC_50_

Paired comparisons of the log LC_50_ values of *B. tabaci* Asia-II-1 showed positive and significant correlations between cypermethrin and three other insecticides like deltamethrin (r = 0.952, P < 0.1), triazophos (r = 0.988, P < 0.05) and imidacloprid (r = 0.995, P < 0.05). For Asia-II-1, a significant positive correlation was observed between deltamethrin and two other insecticides, monocrotophos (r = 0.994, P < 0.05) and imidacloprid (r = 0.979, P < 0.1). Among the Asia-II-1 populations, significant correlation was observed between triazophos and two other neonicotinoids like imidacloprid (r = 0.967, P < 0.1) and thiamethoxam (r = 0.982, P < 0.1). Further, paired comparisons of the log LC_50_ values for the insecticides showed positive and significant correlations between triazophos and imidacloprid (r = 0.911, P < 0.05) within the Asia-I populations of *B. tabaci*. Additionally, a negative correlation was found between chlorpyrifos and most other evaluated insecticides for both the Asia-I and Asia-II-1 populations (Tables 3 and 4).

### Control failure likelihood

The analysis of potential control failure likelihood^54^,^55^ was done by extrapolation of resistance dataset generated in this investigation (Table 5). The analytical test detected possible cases of control failures for both the pyrethroids for all the field populations of *B. tabaci* barring PUSA, with the expected mortality (4 to 30% for cypermethrin; <1 to 14% for deltamethrin) at recommended doses were being significantly lower than the lower confidence limits of their estimated LC_50_ values (Table 5). Similarly, this test detected possible control failure for monocrotophos for all the *B. tabaci* populations except PUSA with the expected mortality at the recommended field dose (150 mg L^−1^) being 3 to 21% (Table 5). This test detected possible control failure for triazophos only for the Nadia population of *B. tabaci*, with its expected mortality being significantly lower than the lower confidence limits of LC_50_ at the recommended dose (800 mg L^−1^). No cases of possible control failures were detected for chlorpyrifos against the chosen *B. tabaci* populations. Regarding the neonicotinoids, possible control failure was detected only for imidacloprid, in all the *B. tabaci* populations except PUSA with the estimated mortalities (<1 to 22%) at recommended field dose (35.7 mg L^−1^) were being significantly lower than lower confidence limits of their LC_50_ estimates. Whereas, for thiamethoxam, this test detected a possible control failure only for Sriganganagar, Ludhiana, Amravati and Guntur populations (Table 5).

### Monitoring of resistance in field populations of *B. tabaci*

**Figure 2:**
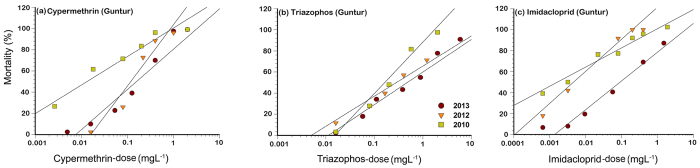
The Mortality response of Guntur population (Asia-I) collected in 2010 to 2013 after the exposure to cypermethrin (**a**), triazophos (**b**), and imidacloprid (**c**). The dose response lines of the each population were drawn using a probit linear model y = αx + β in which α and β are the slope and intercept, respectively. x is the log-transformed dose (mg L^−1^). y is the percent mortality.

**Figure 3:**
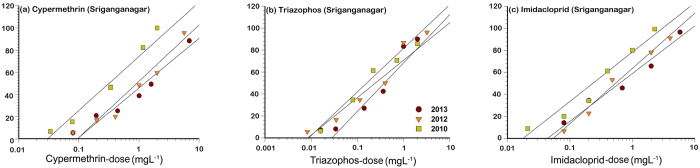
The mortality response of Sriganganagar population (Asia-II-1) collected in 2010 to 2013 after the exposure to cypermethrin (**a**), triazophos (**b**), and imidacloprid (**c**). The dose response lines of the each population were drawn using a probit linear model y = αx + β in which α and β are the slope and intercept, respectively. x is the log-transformed dose (mg L^−1^). y is the percent mortality.

**Figure 4:**
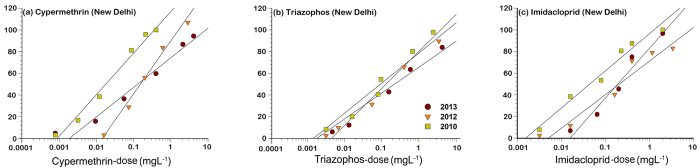
The mortality response of New Delhi population (Asia-II-1) collected in 2010 to 2013 after the exposure to cypermethrin (**a**), triazophos (**b**), and imidacloprid (**c**). The dose response lines of the each population were drawn using a probit linear model y = αx + β in which α and β are the slope and intercept, respectively. x is the log-transformed dose (mg L^−1^). y is the percent mortality.

The field populations of Guntur, New Delhi, and Sriganganagar were used for monitoring the susceptibility of *B. tabaci*. The *B. tabaci* populations were collected from the same fields in three locations during 2010, 2012 and 2013. (The details of collections are summarized in Table 1). The field populations were brought to the laboratory and maintained in separate chambers of insect proof climate control chambers. The mitochondrial cytochrome oxidase 1 sequence analyses revealed that the field populations from the three geographic locations belonged to the same genetic group throughout the course of the investigation with New Delhi and Sriganganagar belonged to Asia-II-1, while, Guntur population was assigned to Asia-I. Changes in dose-mortality responses to imidacloprid, triazophos, and cypermethrin were estimated in the three field populations of *B. tabaci* during 2010 to 2013 for examining dynamics of resistance (Tables 6, 7 and 8; Figs 2, 3 and 4). Substantial variation in dose responses of these three populations to the selected OP, pyrethroid, and neonicotinoid insecticides during 2010–2013 was noticed. Significant loss in susceptibility to imidacloprid in Guntur population was reflected by the increase in LC_50_ value from 11 mg L^–1^ (in 2010) to 130 mg L^–1^ (in 2013) resulting in the 11 fold rise in the resistance ratio from 2010 to 2013. Substantial variation in response to cypermethrin in this population was revealed by the increase in LC_50_ values from 25 mg L^–1^ in 2010 to 261 mg L^–1^ in 2013. This South Indian *B. tabaci* population also showed a threefold loss in susceptibility to triazophos during 2010 to 2013 as indicated by the LC_50_ value of 636 mg L^–1^ in 2013 compared to the baseline LC_50_ value of 167 mg L^–1^ in 2010 (Table 6).

Although the Sriganganagar population was found to be the least susceptible to cypermethrin (LC_50_ = 1362 mg L^–1^) and imidacloprid (LC_50_ = 901 mg L^–1^) as per dose- response analysis in 2013, there was only threefold rise in the resistance ratio from the baseline susceptibility of these compounds in 2010 (Table 7). The New Delhi population had also shown the substantial loss in susceptibility to cypermethrin from 2010 to 2013 as denoted by the sixfold rise in resistance ratio from 2010 to 2013 and this population also showed about the threefold rise in resistance ratios to triazophos and imidacloprid during 2013 compared to the baseline LC_50_ estimates generated during 2010 (Table 8).

## Discussion

The present study is significant because it gives a summary of the current levels of insecticide resistance expressed by *B. tabaci* populations belonging to Asia-I, Asia-II-1, and Asia-II-7, drawn across geographical areas of India. Susceptibility of these populations was varied with Asia-II-7 being the most susceptible, while Asia-I and Asia-II-1 populations were showing significant resistance to the selected organophosphate, pyrethroid and neonicotinoid insecticides.

Worldwide, new and novel chemistries have been employed for the control of sucking pests. However, older chemistries are continued to be in use in India, because they are less expensive. The results of our survey in major cotton growing regions in India have also proved this point. The dose response analysis (Table 2) suggests the development of significant resistance to monocrotophos and to a lesser extent to triazophos and chlorpyrifos in Asia-I and Asia-II-1 genetic groups of *B. tabaci* across geographical areas of India. Compared to an earlier report, there has been a significant increase in the levels of resistance to these OP compounds in the contemporary populations of *B. tabaci*^12^. Higher intensity of the insecticides use (frequency, dose, space) leads to genetically based resistances in insects over time^56^. Very high levels of resistance to monocrotophos noticed in this study in Indian *B. tabaci* populations, with a magnitude of resistance recorded being higher than ever before, could be attributed to the large scale use of this OP compound by the Indian farmers^57^. Similarly, high levels of resistance to triazophos noticed in the *B. tabaci* from Nadia could also be attributed to long term exposure of this *B. tabaci* population to triazophos. Increased frequency of insecticide usage on vegetable crops has been documented in this region^58^. Varying levels of resistance to triazophos has earlier been recorded in Asian genetic groups of *B. tabaci* from India (resistance ratio = 3)^10^, Pakistan (resistance ratio = 42)^37^ and in MEAM 1 genetic group of *B. tabaci* from Turkey (resistance ratio = 310)^38^. Incipient resistance to chlorpyrifos observed in this study is comparable to the reports on the occurrence of 14 fold resistance to this compound in Asian genetic groups of *B. tabaci* from Pakistan^37^.

Low to moderate (resistance ratios ranged from 5 to 45 fold) resistances to cypermethrin have earlier been reported in *the B. tabaci* populations (which may be belonged to Asia-I considering reports of the predominance of Asia-I in this region^53^) from southern India^12^. Our data has shown a considerable increase in the level of resistance to cypermethrin in the contemporary *B. tabaci* Asia-I and Asia-II-1 populations in India compared to the earlier records^12^. Especially, Ludhiana and Sriganganagar locations from northern India recorded a high level of resistance to pyrethroids (Table 2). It may be pertinent to note that this region has been an endemic area of cotton leaf curl disease vectored by *B. tabaci*. We hypothesize that regular outbreaks of cotton leaf curl disease and significantly increased usage of insecticides, including pyrethroids for controlling the vector, could have triggered strong selection pressure for resistance development in these *B. tabaci* populations. The increased use of pyrethroids was found to be one of the factors linked to the recent outbreak of whitefly in cotton belts of Punjab province of India during 2015^13^. Resistance to pyrethroids has been documented in Asia-I^35^, MEAM 1^19^,^21^,^24^,^39^,^59^,^60^, and MED^17^,^18^,^19^,^38^,^61^,^62^ genetic groups of *B. tabaci* across the world.

Significant resistance to imidacloprid recorded in this study could be attributed to the long term exposure of this compound in the cotton ecosystem of this country. Since the inception of commercial *Bt* cotton cultivation in India during 2002, every *Bt* cotton seed has been mandatory treated with a seed dressing formulation of imidacloprid, besides the application of foliar sprays of imidacloprid by farmers for control of sucking pests including whitefly on cotton^7^. Consequently, the imidacloprid seed treatment which had earlier conferred protection against sucking pests upto at least 40 to 45 days after sowing (DAS), was later reported to provide protection for only upto 20–25 DAS^63^. Resistance to neonicotinoids has widely been documented in Asia-I^35^, MEAM1^16^,^18^,^19^,^30^,^41^,^64^,^65^ and MED^16^,^33^,^41^,^42^,^44^,^61^,^64^ genetic groups of *B. tabaci* in many Asian, American, European and Mediterranean countries.

Paired comparisons of the log LC_50_ values for the insecticides showed significant positive correlations between OP, Pyrethroid and neonicotinoid compounds and a negative correlation was found between chlorpyrifos and other insecticides evaluated in the *B. tabaci* Asia-I and Asia-II-1 populations (Table 4). In line with the revelation of several earlier works, we speculate the possibility of cross resistance between imidacloprid, OP and pyrethroid compounds. The concurrent occurrence of high levels of resistance to OPs and pyrethroids had been observed in West Africa, Pakistan, and Turkish *B. tabaci* populations^17^,^37^,^38^. Inconsistency in the neonicotinoid cross-resistance pattern has been reported by Prabhaker *et al*.^41^ and by Horowitz *et al*.^30^. Earlier reports from china^66^ and US^43^ have also demonstrated the prevalence of cross resistance between imidacloprid and thiamethoxam in an MEAM genetic group of *B. tabaci*^34^, while, studies with Cyprus populations of *B. tabaci* (MEAM 1 genetic group) revealed the absence of cross resistance between these two neonicotinoid compounds^21^,^67^. Besides target site insensitivity, one or more metabolic resistance mechanisms involving carboxylesterases, cytochrome-P450-dependent monooxygenases, and glutathione S-transferases were implicated in *B. tabaci* resistant to OP, pyrethroid and neonicotinoid insecticides^36^,^37^,^38^,^39^,^40^. It is plausible that Indian *B. tabaci* populations might have evolved multiple resistance mechanisms in response to field application of these insecticides in the past. Therefore, detailed cross resistance studies need to be undertaken in Indian *B. tabaci* populations for devising suitable insecticide resistance management strategies.

Several global studies have documented resistance in *B. tabaci* MED and MEAM 1 genetic groups to different groups of insecticides across the continents^3^. However, there is a limited literature available on the insecticide status of indigenous *B. tabaci* genetic groups of Asia. This study clearly provided the insecticide resistance/susceptibility status of Asian genetic groups like Asia-I, Asia-II-1, and Asia-II-7 against the selected OP, pyrethroid, and neonicotinoid compounds. As insecticide resistance is regarded by some workers as a major driving force for the selection and establishment of specific *B. tabaci* genetic groups in a region^19^,^30^,^31^,^51^, there is a need for regular monitoring of insecticide resistance status in diverse *B. tabaci* genetic groups in India.

Knowledge on the susceptibility level of insect populations from different geographical areas is critical for measuring the trends in temporal and spatial resistance development of *B. tabaci*^45^. Likewise, our studies have established the decrease in susceptibility levels of three *B. tabaci* populations to select OP, pyrethroid, neonicotinoid insecticides during 2010 to 2013 (Table 5) and the trend clearly showed the evolution of significant resistances to these insecticides in North Indian field populations of *B. tabaci*. The recent outbreak of whitefly in the Punjab state of India would appear to be at least partly due to the manifestation of significant resistance development in the field populations of *B. tabaci*^13^.

The higher values of LC_50_ along with the high value of the slopes (Table 2; Figs 2, 3 and 4) may be indicating significant resistance development in Indian field populations of *B. tabaci*. Chilcuit and Tabashnik^68^ proposed that slope was not a good indicator of the genetic variability in susceptible organisms, and further, that genetic variation was not related to the LC_50_ values. However, Hussain *et al*.^69^ opined that the higher inter-population variations in the slopes coupled with high level of resistance to the insecticides indicated the possibility of an existence of qualitatively different resistance mechanisms in field strains of *H. armigera* in Pakistan. Hence, further studies are needed to unravel the biochemical and molecular basis of resistance to these compounds in Indian *B. tabaci* populations.

The potential for control failure of insecticides was estimated by use of analytical tools as described in Silva *et al*.^54^ and Roditakis *et al*.^55^. Our results (Table 5) indicate the likelihood of control failures for insecticides such as monocrotophos, imidacloprid, cypermethrin and deltamethrin at the recommended label rates in the selected field populations. Nevertheless, that it was only an estimate and was not based on a rigorous assessment of actual control efficacy of the said chemicals against the field populations. Our results suggest that the field dose of these chemicals have to be higher than the recommended label rate of Central Insecticides Board and Registration Committee, Government of India, to have effective control of *B. tabaci* at least in these regions. Although insecticide quality is legitimately regulated in India, factors such as poor knowledge on the selection of chemicals by the farmers, use of unscientific tank mixtures and sub-standard application practices exacerbate the problem of control failure of insecticides in field conditions^12^,^63^. Hence, appropriate field tests are needed to verify the bioefficacy of these chemicals at recommended label rates against these populations of *B. tabaci*.

Integrated Pest Management (IPM) has been the overriding principle of plant protection in India and greater emphasis is laid on reducing dependency on chemical control in several crop pests. As our results have shown the widespread development of resistance to OP, pyrethroids and neonicotinoids in the *B. tabaci* genetic groups, Asia-I and Asia-II-1, we emphasize the need for undertaking regular monitoring of insecticide resistance status of different *B. tabaci* genetic groups across India. The management of this pest in India may be strengthened by taking clues from successful global IPM programmes.

Host plant resistance is a major, often preventative measure for managing *B. tabaci.* Studies have shown that pubescent varieties are more preferred by *B. tabaci* as compared with glabrous ones^70^,^71^. Natural defenses in a wild species of cotton, *Gossypium arboretum,* including long trichome or presence of inorganic salts with increased concentration of waxes provide protection against whitefly and cotton leaf curl virus^72^. Increasing the area under indigenous varieties of *G. arboreum* may mitigate the frequent epidemics of whitefly and cotton leaf curl virus especially in Northwestern India.

Rotational scheme of insecticides with different modes of action has been found effective in insecticide resistance management of *B. tabaci* in Israel. Application of pyriproxyfen in cotton during the first month, followed by an additional treatment with buprofezin (if required), do not markedly alter the susceptibility of *B. tabaci* to either compounds or no appreciable increase of resistance to the conventional insecticides^73^. Application of insect growth regulators like pyriproxyfen or buprofezin during the early stage of crop growth is found effective in controlling MEAM 1 genetic group of *B. tabaci* in Arizona, USA, as these insect growth regulator compounds have helped to conserve natural enemies and substantially reduce sprays of broad-spectrum insecticides^74^.

The refuge strategy is mandated by the regulatory authorities worldwide to manage the evolution of resistance in bollworms targeted by *Bt* cotton. Simulation analysis has shown the effectiveness of this strategy in delaying insecticide resistance in MEAM 1 genetic group of *B. tabaci*^75^. Although *B. tabaci* is polyphagous, the cotton refuges have been particularly found more useful in delaying insecticide resistance development in *B. tabaci*^76^.

Therefore, a comprehensive, integrated pest management and insecticide resistance management strategies, including identification of whitefly resistant *Bt* hybrids and *G. arboreum* genotypes, rotation of conventional insecticides with novel molecules including insect growth regulator (IGR) compounds, use of sticky traps and exploitation of native biological control agents will augur the sustainable management of *B. tabaci* in the Indian subcontinent.

## Methods

### Whitefly collection, rearing, and maintenance

The field populations of *B. tabaci* were collected from seven locations across eastern, central, southern and northern regions of India. Geographically, these locations fall under five agro-climatic zones of India (India has 15 agro-climatic zones). Uniform whitefly infestation pattern and easy accessibility encouraged us to select these regions for the collection of *B. tabaci* populations. To generate adequate information on the use of insecticide on cotton and vegetable fields, Knowledge-Attitude-Practice (KAP) surveys were conducted in 2010 to 2013 in the study sites by following the protocol used by Yadouleton *et al*.^77^. Briefly, ten farmers in each locality were interviewed by using a semi-structured questionnaire focussing on the insecticide application pattern in the farms. Further, qualitative data were collected through direct observations and group discussions. The descriptions of the collection sites, the period of the collections, genetic group identity of *B. tabaci* populations and the background information on cropping pattern and insecticide application details are presented in Table 1. The exact locations of the collection sites are presented in Fig. 1.

While collecting, standard procedure was followed by walking in ‘Z’ mode at a minimum of two-hectare blocks of the crops. Insects were collected using an aspirator during early morning along with infested leaves containing the nymphs and pupae. The insects were transported to the laboratory in ventilated cages containing leaflets inserted into wet sponges. Infested leaflets were kept in cages for the emergence of fresh adults. The taxonomic identity of *B. tabaci* species complex was confirmed by examining the insects under a light microscope using the keys of Martin *et al*.^78^,^79^. These populations had been raised on insecticide-free cotton plants (*G. hirsutum.*) at temperatures of 27 ± 2 °C, photoperiod of 14:10 h (Light:Dark) and relative humidity of 60–70% in quarantined insect growth chambers. These populations were maintained as large colonies for five generations without insecticide selection prior to the current bioassays.

### Genetic group determination

The genetic group identity of *B. tabaci* field population was examined by random sampling of 10 adults for each population using the PCR amplification of mitochondrial cytochrome oxidase 1 gene and sequencing technique as described in Dinsdale *et al*.^80^. DNA extraction was performed by using single adult females with the DNeasy Blood and Tissue Kit (Qiagen GmbH, Hilden, Germany). Sequencing was done by outsourcing with SciGenom Labs (Cochin, Kerala, India). Genetic group determination was carried out by the direct sequence comparisons using the web-based Basic Local Alignment Search Tool algorithm of NCBI (https://blast.ncbi.nlm.nih.gov/Blast.cgi). The genetic group identity was further confirmed by the phylogenetic and molecular evolutionary analysis with well-assigned homologous sequences of the *B. tabaci* genetic groups from the consensus sequence database using MEGA version 6 1,81.

### Insecticides

Purity analyzed technical grade insecticides such as triazophos (60.9%), monocrotophos (99%) and chlorpyrifos (60%); cypermethrin (99.3%); imidacloprid (96.4%), thiamethoxam (98%) and deltamethrin (98%) were procured from the insecticide manufacturers. These insecticides were selected, as they represented the OPs, pyrethroids and neonicotinoids concurrently used for control of whitefly in the respective regions where the whitefly populations were collected (Table 1). These compounds also had the label claim for efficacy against whiteflies as per the registered use of pesticides with Central Insecticides Board and Registration Committee, Government of India as on date (http://www.cibrc.nic.in).

### Bioassay

For assessing the insecticide toxicity to *B. tabaci*, a modified leaf dip bioassay method of Insecticide Resistance Action Committee was followed^82^. The stock solutions of technical grade insecticides were prepared in acetone, with serial dilutions in deionized water containing 0.1 g L^–1^ of non-ionic wetting agent Triton X-100. Cotton leaves with petiole, collected from the fifteen to twenty-five days old seedlings were immersed in the serially diluted insecticide solutions for 20 sec; then allowed to air dry on paper towel and kept on agar slants (2%) in Petri plates (90 × 15 mm). Leaves dipped in only diluents served as the untreated control. The adults were briefly anesthetized using CO_2_ and transferred in batches of 15–20 onto the treated leaves. The plates were sealed with ventilated lids. All such assays were replicated five times for a minimum of five concentrations for each insecticide. All treatments were placed in an insect rearing room with the temperature, photoperiod, and RH conditions as mentioned earlier. As the mortality rate was too low at earlier hours in some of the doses, observations were taken for an extended period 96 h as described by Gorman *et al*.^16^. The adult insect was considered to be dead if no coordinated movement or deficient response to external stimulus (i.e. when gently probed with a fine paintbrush) was observed under the light microscope. Mortality was estimated by counting the total number of dead and live insects.

### Monitoring insecticide susceptibility in *B. tabaci* populations

To compare the changes in susceptibility of *B. tabaci* populations over time, the populations were collected from same cotton fields located in New Delhi, Sriganganagar, and Guntur (the location details and time of collections are furnished in Table 1). The collection, maintenance and genetic group identity of these *B. tabaci* field populations were done as described in the earlier section. Dose responses were generated for three insecticides *viz*., triazophos, cypermethrin, and Imidacloprid during 2010, 2012 and 2013. The details of field populations and their genetic group identities are presented in Table 1.

### Data analysis

The mortality data were corrected according to Abbott’s formula^83^. The LC_50_ and LC_90_ values, 95% confidence limits, standard errors, the slopes of the regression lines and χ^2^ significance tests, were estimated by probit analysis^84^ using PoloPlus 2.0 software (LeOra Software, California, United States). The resistance ratios were calculated by the “lethal ratio test” and were considered significant when the confidence limits at 95% did not include the value one as proposed by Robertson *et al*.^85^. The resistance ratio for each insecticide was calculated with reference to a population of the same genetic group with the lowest LC_50_ or LC_90_ 17,35. More specifically, resistance ratio = LC_50_ or LC_90_ of each population was divided by the LC_50_ or LC_90_ of the most susceptible population within Asia-I or Asia-II-1. In the absence of a characterized susceptible strain, the actual resistance level could be underestimated by mere comparisons within the genetic groups. Therefore, additional resistance ratios were also computed for each insecticide with reference to the most susceptible *B. tabaci* population (belonging to Asia-II-7 collected from New Delhi and designated as PUSA) to demonstrate the magnitude of resistance development in Indian *B. tabaci* populations in the present dataset.

Pearson correlation coefficient (r) test was applied to test the significance of pairwise comparison between the different attributes (log LC_50_). The correlation analysis was conducted using SPSS version 16.0. (SPSS Inc. Chicago, Illinois, USA).

The potential for the likelihood of control failure of insecticides was estimated on the basis of Silva *et al*.^54^ and Roditakis *et al*.^55^. As LC_50_ is the most reliable point of comparison for dose response regressions^85^, the 50% mortality was used as a threshold value between control success and failure. The estimated LC_50_ and 95% confidence limits were compared with the maximum recommended field rate by Central Insecticides Board and Registration Committee, Government of India. The maximum recommended label rates for the tested insecticides in India for whitefly or sucking pest were: cypermethrin 100 mg L^−1^, deltamethrin 16.67 mg L^−1^, triazophos 800 mg L^−1^, monocrotophos 150 mg L^−1^, chlorpyrifos 250 mg L^−1^, imidacloprid 35.7 mg L^−1^ and thiamethoxam 66.67 mg L^−1^. Briefly, the mortality expressed at the maximum recommended rate was estimated by using the PriProbit Software 1.5^86^. The mortality achieved by the label rate would be considered to be significantly lower than 50% when the lower 95% confidence limits of the LC_50_ were found to be higher than the recommended rate.

## Additional Information

**How to cite this article**: Naveen, N. C. *et al*. Insecticide resistance status in the whitefly, *Bemisia tabaci* genetic groups Asia-I, Asia-II-1 and Asia-II-7 on the Indian subcontinent. *Sci. Rep.* **7**, 40634; doi: 10.1038/srep40634 (2017).

**Publisher's note:** Springer Nature remains neutral with regard to jurisdictional claims in published maps and institutional affiliations.

## Figures and Tables

**Table 1:** Survey locations and descriptions of *B. tabaci* populations.

Collection descriptions							
Populations	Geographic origin (Agro-climatic zone - States)	GPS coordinates	Year	Common insecticides used for control of whitefly in the farms	Host plant and stage of collection	Adjacent crops	Identification (Genetic group)
New Delhi	Trans Gangetic Plains Region -Delhi	28° 38′ 5.940″ N 77° 09′ 6.750″ E	2010	triazophos, chlorpyrifos, imidacloprid and thiamethoxam	cotton (boll formation stage)	cotton and vegetables	Asia-II-1
			2012	imidacloprid and thiamethoxam			Asia-II-1
			2013	chlorpyrifos, imidacloprid and thiamethoxam			Asia-II-1
Sriganganagar	Western Dry Region- Rajasthan	29° 55′ 12″ N 73° 52′ 48″ E	2010	triazophos, monocrotophos, imidacloprid, thiamethoxam, thiodicarb, mixtures of chlorpyrifos with cypermethrin	cotton (square formation stage)	cotton, vegetables and sugar cane	Asia-II-1
			2012	triazophos, monocrotophos, fipronil, mixtures of chlorpyrifos with cypermethrin, indoxacarb with acetamiprid			Asia-II-1
			2013	triazophos, fipronil monocrotophos, imidacloprid, thiamethoxam, thiodicarb			Asia-II-1
Ludhiana	Trans Gangetic Plains Region-Punjab	30° 36′ 0.338″ N 74° 47′ 41.719″ E	2012	triazophos, monocrotophos, imidacloprid, thiamethoxam, fipronil, mixtures of chlorpyrifos with imidacloprid and deltamethrinwith triazophos	cotton (square formation stage)	cotton, vegetables and maize	Asia-II-1
Amravati	Western Plateau and Hills region-Maharashtra	20° 55′ 32.999 N 77° 45′ 52.999″ E	2013	triazophos, chlorpyrifos, monocrotophos, thiamethoxam, imidacloprid and thiamethoxam	cotton (square formation stage)	cotton and soybean	Asia-I
Khandwa	Western Plateau and Hills region-Madhya Pradesh	21° 49′ 32.640″ N 76° 21′ 9.256″ E	2012	triazophos, acephate, monocrotophos, imidacloprid, thiodicarb and endosulfan	cotton (early stage of boll formation)	cotton, soybean and groundnut	Asia-I
Nadia	Lower Gangetic Plains Region -West Bengal	23° 39′ 35.558″ N 88° 24′ 5.774″ E	2012	triazophos, indoxacarb, chlorpyrifos, acephate, monocrotophos, imidacloprid and mixtures of deltamethrin withtriazophos	brinjal (late stage of flowering)	vegetables	Asia-I
Guntur	East Coast Plains and Hills Region -Andhra Pradesh	16° 17′ 54.636″ N 80° 26′ 1.129″ E	2010	chlorpyrifos, endosulfan, fipronil, imidacloprid, thiamethoxam, mixtures of chlorpyrifos with imidacloprid and indoxacarb with acetamiprid	cotton (early stage of boll formation)	cotton, vegetables, maize, mung bean and tobacco	Asia-I
			2012	imidacloprid and thiamethoxam			Asia-I
			2013	triazophos, chlorpyrifos imidacloprid, indoxacarb, acetamiprid			Asia-I
PUSA	Trans Gangetic Plains Region -Delhi	28° 38′ 5.430″ N 77° 09′ 8.410″ E	2012	—	cotton(boll formation stage)	cotton and vegetables	Asia-II-7

**Table 2:** Log-dose (mg L^–1^) probit mortality data of *B. tabaci* populations tested.

Populations	Genetic group	N	Slope ± SE	χ^2^ a df^b^	LC_50_ (CL 95%)	RR_50_^c^ (CL 95%)	RR_50_^d^ (CL 95%)	RR_50_^e^ (CL 95%)	LC_90_ (CL 95%)	RR_90_^c^ (CL 95%)	RR_90_^d^ (CL 95%)	RR_90_^e^ (CL 95%)
**Cypermethrin**												
PUSA	Asia-II-7	197	2.06 ± 0.35	9.64 (4)	10 (4–15)	1.00	—	—	43 (27–140)	1.00	—	—
New Delhi	Asia-II-1	290	1.51 ± 0.12	12.82* (5)	194 (104–350)	19.40 (12.58–31.93)	1.00	—	1840 (924–5733)	42.80 (23.72–76.33)	1.00	—
Ludhiana	Asia-II-1	218	1.94 ± 0.46	4.60 (5)	780 (368–1150)	78.00 (45.28–144.04)	4.03 (2.22–7.31)	—	3558 (2327–8899)	82.80 (42.35–159.86)	1.93 (0.93–4.01)	—
Sriganganagar	Asia-II-1	290	1.32 ± 0.12	8.20 (5)	1362 (741–3734)	136.20 (86.36–230.06)	7.03 (4.22–11.71)	—	10518 (3806–16623)	244.60 (101–585.70)	5.72 (2.26–14.48)	—
Khandwa	Asia-I	275	1.98 ± 0.34	5.07 (4)	701 (236–1155)	70.10 (43.84–120.01)	—	3.00 (1.67–5.18)	3110 (1857–10484)	72.32 (41.45–124.75)	—	4.69 (2.70–8.17)
Amravati	Asia-I	211	1.34 ± 0.21	3.22 (4)	238 (157–362)	23.80 (14.77–41.17)	—	1.00	662 (394–2600)	15.32 (9.259–25.345)	—	1.00
Nadia	Asia-I	237	2.24 ± 0.31	11.16* (5)	255 (111–439)	25.50 (17.18–40.68)	—	1.07 (0.65–1.77)	949 (536–3671)	22.00 (13.01–37.03)	—	1.43 (0.85–2.43)
Guntur	Asia-I	238	2.89 ± 0.51	7.08 (5)	261 (131–383)	26.10 (17.74–41.20)	—	1.10 (0.67–1.80)	725 (482–1909)	16.86 (10.23–27.44)	—	1.09 (0.67–1.80)
**Deltamethrin**												
PUSA	Asia-II-7	298	2.19 ± 0.15	10.87 (6)	10 (2–22)	1.00			153 (66–265)	1.00	—	—
New Delhi	Asia-II-1	261	1.48 ± 0.15	9.88 (5)	120 (62–236)	12.00 (6.349–21.741)	1.00	—	877 (401–3835)	5.73 (2.57–12.80)	1.00	—
Ludhiana	Asia-II-1	214	4.81 ± 0.99	1.89 (3)	760 (480–1032)	76.00 (39.99–138.02)	6.33 (3.87–10.33)	—	1402 (1032–2086)	9.16 (4.68–17.92)	1.60 (0.85–3.03)	—
Sriganganagar	Asia-II-1	254	2.26 ± 0.30	1.08 (5)	715 (519–960)	71.50 (38.63–126.65)	5.95 (3.77–9.41)	—	2639 (1842–4509)	17.25 (8.32–35.72)	3.01 (1.50–6.05)	
Khandwa	—	—	—	—	—	—	—	—	—	—	—	—
Amravati	Asia-I	228	1.33 ± 0.14	5.07 (4)	128 (71–229)	12.80 (6.76–23.33)	—	1.00	1168 (561–4298)	7.63 (3.33–17.51)	—	1.00
Nadia	—	—	—	—	—	—	—	—	—	—	—	—
Guntur	Asia-I	236	1.98 ± 0.17	5.92 (4)	242 (52–675)	24.20 (10.82–51.68)	—	2.00 (0.95–3.75)	1204 (785–3196)	7.87 (9.18–69.48)	—	1.03 (0.63–9.01)
**Triazophos**												
PUSA	Asia-II-7	230	1.50 ± 0.17	6.87 (4)	53 (24–102)	1.00	—		392 (183–1794)	1.00		—
New Delhi	Asia-II-1	228	2.22 ± 0.37	5.57 (5)	324 (199–517)	6.11 (3.80–9.86)	1.00	—	1219 (708–4113)	3.11 (1.46–6.63)	1.00	—
Ludhiana	Asia-II-1	231	2.86 ± 0.46	0.35 (5)	428 (319–549)	8.10 (5.20–12.64)	1.32 (0.88–1.99)	—	1298 (890–1942)	3.31 (1.56–5.85)	1.06 (0.55–1.88)	—
Sriganganagar	Asia-II-1	336	2.53 ± 0.34	3.43 (4)	525 (402–647)	9.91 (6.48–15.25)	1.62 (1.00–2.40)	—	1683 (1314–2422)	4.29 (2.33–7.92)	1.38 (0.75–2.54)	—
Khandwa	Asia-I	264	2.76 ± 0.55	8.98 (5)	445 (131–672)	8.40 (4.30–14.32)	—	1.00	2875 (1615–8758)	7.33 (3.35–16.08)	—	1.00
Amravati	Asia-I	203	1.50 ± 0.18	4.20 (5)	532 (382–757)	10.04 (6.15–16.49)	—	1.20 (0.71–2.31)	3773 (2260–8205)	9.63 (4.23–21.97)	—	1.31 (0.56–3.06)
Nadia	Asia-I	239	2.46 ± 0.50	5.91 (6)	1429 (1071–1927)	27.00 (17.22–42.49)	—	3.21 (1.98–6.00)	4730 (3104–11650)	12.07 (5.51–26.50)	—	1.65 (0.73–3.71)
Guntur	Asia-I	330	1.63 ± 0.22	9.54 (6)	636 (358–1016)	12.00 (7.50–19.28)	—	1.43 (0.87–2.71)	3847 (2105–13290)	9.81 (4.69–20.58)	—	1.34 (0.62–2.88)
**Monocrotophos**												
PUSA	Asia-II-7	231	1.58 ± 0.21	4.79 (4)	88 (42–154)	1.00	—	—	298 (169–721)	1.00	—	—
New Delhi	Asia-II-1	221	1.48 ± 0.16	2.92 (4)	528 (370–730)	6.00 (3.61–10.09)	1.00	—	1941 (1363–3057)	6.51 (3.63–11.78)	1.00	—
Ludhiana	Asia-II-1	306	1.67 ± 0.33	4.50 (6)	2114 (1536–3079)	24.02 (14.57–40.06)	4.00 (2.50–6.40)	—	6732 (4256–17144)	22.59 (10.69–47.73)	3.47 (1.67–7.22)	—
Sriganganagar	Asia-II-1	239	1.87 ± 0.17	3.81 (5)	1710 (1094–3075)	19.43 (10.36–36.86)	3.24 (1.77–5.93)	—	7833 (4117–21842)	26.31 (10.64–65.00)	4.04 (1.65–9.85)	—
Khandwa	Asia-I	244	1.60 ± 0.44	3.30 (4)	2480 (1549–4133)	28.18 (16.00–50.24)	—	2.94 (1.56–5.56)	8279 (4741–40222)	27.80 (11.15–69.21)	—	2.48 (0.90–6.82)
Amravati	Asia-I	210	1.41 ± 0.29	3.85 (5)	843 (464–1308)	9.58 (5.21–17.80)	—	1.00	3337 (2054–8098)	11.20 (5.32–23.58)	—	1.00
Nadia	Asia-I	220	1.36 ± 0.34	1.97 (4)	3934 (2323–9720)	44.70 (21.82 –92.60)	—	5.0 (2.15–10.12)	9537 (4933–48445)	32.00 (16.08–50.78)	—	2.86 (1.70–13.55)
Guntur	Asia-I	263	1.35 ± 0.24	0.70 (4)	1478 (934–2288)	16.80 (9.40–30.04)	—	1.75 (0.93–3.32)	6179 (3691–15510)	20.74 (9.49–45.32)	—	1.85 (0.76–4.53)
**Chlorpyrifos**												
PUSA	Asia-II-7	221	1.70 ± 1.78	0.03 (4)	12 (11– 14)	1.00	—	—	16 (15–19)	1.00	—	—
New Delhi	Asia-II-1	223	1.57 ± 0.17	8.78 (5)	201 (111–406)	16.80 (11.26–23.25)	1.5 (1.04–2.50)	—	1320 (599–5971)	82.50 (43.88–147.40)	3.27 (1.33–5.42)	—
Ludhiana	Asia-II-1	246	1.92 ± 0.19	2.23 (4)	137 (99–191)	11.42 (8.97–17.27)	1.00	—	404 (212–1192)	25.25 (16.268–37.22)	1.00	—
Sriganganagar	Asia-II-1	208	2.19 ± 0.32	3.57 (4)	163 (117–218)	13.58 (9.57–17.99)	1.19 (0.89–1.94)	—	626 (440–1069)	39.13 (24.55–59.18)	1.55 (0.73–2.24)	—
Khandwa	Asia-I	183	2.01 ± 0.24	4.44 (4)	56 (33–88)	4.70 (3.263–6.245)	—	1.00	245 (147–572)	15.31 (9.66–23.04)	—	1.00
Amravati	Asia-I	220	1.64 ± 0.20	7.74 (5)	220 (129–412)	18.33 (12.46–24.98)	—	3.91 (2.47–6.19)	1326 (638–5374)	82.90 (44.40–146.95)	—	5.42 (2.63–11.14)
Nadia	Asia-I	189	2.34 ± 0.29	6.34 (4)	118 (63–189)	9.83 (7.04–12.69)	—	2.09 (1.37–3.19)	416 (251–1054)	26.00 (17.49–36.77)	—	1.70 (0.98–2.94)
Guntur	Asia-I	228	2.17 ± 0.40	5.03 (5)	309 (173–439)	25.75 (18.06–34.20)	—	5.51 (3.55–8.54)	1204 (785–3196)	75.25 (46.45–115.10)	—	4.92 (2.66–9.05)
**Imidacloprid**												
PUSA	Asia-II-7	214	1.65 ± 0.15	3.72 (6)	52 (19–80)	1.00	—	—	601 (247–386)	1.00	—	—
New Delhi	Asia-II-1	247	1.52 ± 0.22	3.64 (5)	178 (122–296)	3.42 (1.68–6.08)	1.00	—	1871 (569–8436)	3.11 (816–6391)	1.00	—
Ludhiana	Asia-II-1	416	2.00 ± 0.11	9.03 (5)	664 (307–2016)	12.77 (8.98–40.08)	3.73 (2.95–11.87)	—	5032 (1543–8209)	8.37 (4.35–15.64)	2.69 (1.95–9.87)	—
Sriganganagar	Asia-II-1	221	2.11 ± 0.20	3.14 (5)	901 (581–1958)	17.33 (8.74–39.74)	5.06 (2.86–11.78)	—	5517 (2542–13506)	9.18 (2.89–37.94)	2.95 (2.58– 26.75)	—
Khandwa	Asia-I	273	2.23 ± 0.36	7.00 (5)	175 (99–257)	3.37 (1.93–5.95)	—	1.34 (0.88–2.06)	857 (412–1940)	1.41 (0.47–2.53)	—	1.10 (0.48–1.36)
Amravati	Asia-I	232	1.88 ± 0.25	7.91 (4)	170 (120–338)	3.30 (1.80–6.01)	—	1.30 (0.81–2.10)	815 (413–6290)	1.36 (0.61–3.03)	—	1.05 (0.58–1.91)
Nadia	Asia-I	266	1.72 ± 0.22	3.52 (3)	956 (632–1780)	18.40 (10.55–32.40)	—	7.33 (4.80–11.20)	5322 (2534–11001)	8.86 (3.45–22.74)	—	6.87 (3.16–14.94)
Guntur	Asia-I	261	1.37 ± 0.13	2.78 (4)	130 (95–183)	2.50 (1.39–4.57)	—	1.00	774 (368–2881)	1.290 (0.53–3.13)	—	1.00
**Thiamethoxam**												
PUSA	Asia-II-7	251	1.55 ± 0.17	7.58 (4)	26 (7–40)	1.00	—	—	233 (106–267)	1.00	—	—
New Delhi	Asia-II-1	353	1.83 ± 0.17	8.25 (5)	73 (45–126)	2.81 (1.689–4.602)	1.00	—	358 (212–821)	1.53 (0.80–2.94)	1.00	—
Ludhiana	Asia-II-1	290	1.50 ± 0.15	7.37 (4)	109 (76–252)	4.19 (2.434–7.158)	1.50 (1.00–2.22)	—	915 (358–1437)	3.93 (1.84–8.37)	2.56 (1.31–5.00)	—
Sriganganagar	Asia-II-1	220	1.56 ± 0.15	7.54 (4)	194 (93–442)	7.5 (4.22–13.18)	2.67 (1.73–4.14)	—	2036 (762–5407)	8.74 (3.62–21.04	5.69 (2.55–12.71)	—
Khandwa	Asia-I	264	1.90 ± 0.25	3.16 (4)	23 (17–31)	0.88 (0.52–1.50)	—	1.00	111 (76–196)	0.48 (0.24–0.95)	—	1.00
Amravati	Asia-I	332	1.92 ± 0.13	5.91 (5)	176 (95–302)	6.77 (3.77–12.17)	—	7.69 (4.70–12.60)	1128 (480–8879)	4.84 (2.38–9.83)	—	10.18 (5.24–19.79)
Nadia	—	—	—	—	—	—	—		—	—	—	
Guntur	Asia-I	276	2.60 ± 0.40	2.85 (4)	179 (119–222)	6.88 (4.18–11.24)	—	7.78 (5.32–11.37)	559 (415–915)	2.40 (1.26–4.55)	—	5.04 (2.79–9.11)

^a^Chi-square test for linearity of the dose–mortality response: ***P < 0.001, **P < 0.01, *P < 0.05.

^b^Degrees of freedom. Resistance ratios (RR) with 95% confidence limits indicating the fold-difference for each population in comparison to the most susceptible population at LC_50_ and LC_90_. Confidence limits that include 1.0 indicate no significant difference from the susceptible population (Lethal ratio test-Robertson *et al*.^85^).

^c^RR = Asia-I or Asia-II-1 populations divided by most susceptible Asia-II-7 population.

^d^RR = Asia-II-1 populations divided by most susceptible Asia-II-1 population.

^e^RR = Asia-I populations divided by most susceptible Asia-I population.

**Table 3:** Correlation coefficients of pairwise comparisons between the log LC_50_ values of the evaluated insecticides towards Asia-II-1 *B. tabaci* populations^a^.

	Cypermethrin	Deltamethrin	Triazophos	Monocrotophos	Chlorpyrifos	Imidacloprid
Deltamethrin	0.952^[0.099]*^					
Triazophos	0.988^[0.049]**^	0.894^[0.148]ns^				
Monocrotophos	0.912^[0.135]ns^	0.994^[0.036]**^	0.838^[0.184]ns^			
Chlorpyrifos	−0.731^[0.478]ns^	−0.904^[0.140]ns^	−0.617^[0.288]ns^	−0.947^[0.104]ns^		
Imidacloprid	0.995^[0.033]**^	0.979^[0.066]*^	0.967^[0.082]*^	0.949^[0.102]ns^	−0.790^[0.206]ns^	
Thiamethoxam	0.941^[0.110]ns^	0.792^[0.209]ns^	0.982^[0.061]*^	0.718^[0.245]ns^	−0.456^[0.349]ns^	0.90^[0.143]ns^

^a^Correlation significance ***P < 0.01, **P < 0.05, *P < 0.1, ns: Not significant.

**Table 4:** Correlation coefficients of pairwise comparisons between the log LC_50_ values of the evaluated insecticides towards Asia-I *B. tabaci* populations^a^.

	Cypermethrin	Triazophos	Monocrotophos	Chlorpyrifos
Triazophos	−0.522^[0.239]ns^			
Monocrotophos	0.328^[0.336]ns^	0.627^[0.186]ns^		
Chlorpyrifos	−0.842^[0.079]*^	0.084^[0.458]ns^	−0.623^[0.189]ns^	
Imidacloprid	−0.238^[0.381]ns^	0.911^[0.044]**^	0.746^[0.127]ns^	−0.288^[0.356]ns^

^a^Correlation significance *** P < 0.01, **P < 0.05, *P < 0.1, ns: Not significant.

**Table 5:** Estimated percentage mortality of the *B. tabaci* populations extrapolated from assay mortalities compared to the maximum recommended label rate of Indian legislation (CIBRC)^a^.

Groups	Insecticides	PUSA	New Delhi	Ludhiana	Sriganganagar	Khandwa	Amravati	Nadia	Guntur
Pyrethroids	Cypermethrin	100	22*	4*	5*	5*	30*	18*	10*
	Deltamethrin	63	12*	<1*	<1*	—	14*	—	14*
Organophosphates	Triazophos	98	81	79	68	67	61	27*	57
	Monocrotophos	65	21*	3*	9*	3*	15*	3*	9*
	Chlorpyrifos	100	56	80	66	90	54	78	42
Neonicotinoids	Imidacloprid	44	17*	14*	5*	6*	10*	<1*	22*
	Thiamethoxam	100	49	40*	29*	82	35*	—	15*

^a^Maximum recommended field rates for the tested insecticides in India by Central Insecticide Board of Registration Committee (CIBRC) for whitefly or sucking pest were: cypermethrin 100 mg L^−1^, deltamethrin 16.67 mg L^−1^, triazophos 800 mg L^−1^, monocrotophos 150 mg L^−1^, chlorpyrifos 250 mg L^−1^, imidacloprid 35.7 mg L^−1^ and thiamethoxam 66.67 mg L^−1^.

^*^Mortality significantly lower than 50% because the recommended field rate is lower than the lower threshold of the insecticide LC_50_ confidence limits of the population (see the Table 2).

**Table 6:** Log-dose (mg L^–1^) probit model fitted to mortality data of Guntur *B. tabaci* populations collected during 2010 to 2013.

Population	Sampling year	Genetic group	N	Slope ± SE	χ^2^ a (df)^b^	LC_50_(CI 95%)	RR^c^
**Cypermethrin**							
Guntur	2010	Asia-I	284	1.32 ± 0.17	9.54 (4)	25 (5–57)	1.00
Guntur	2012	Asia-I	253	2.27 ± 0.26	2.58 (4)	127 (96–165)	5.16 (3.05–8.73)
Guntur	2013	Asia-I	238	2.89 ± 0.51	7.08 (5)	261 (131–383)	10.60 (6.20–18.01)
**Triazophos**							
Guntur	2010	Asia-I	245	2.14 ± 0.30	4.22 (4)	167 (99–257)	1.00
Guntur	2012	Asia-I	246	1.26 ± 0.23	2.86 (4)	321 (204–528)	1.92 (1.11–3.31)
Guntur	2013	Asia-I	330	1.63 ± 0.22	9.54 (6)	636 (358–1016)	3.78 (2.46–5.85)
**Imidacloprid**							
Guntur	2010	Asia-I	330	1.10 ± 0.15	10.90 (5)	11 (2–24)	1.00
Guntur	2012	Asia-I	254	1.90 ± 0.25	2.73 (4)	26 (18–36)	2.36 (1.31–4.61)
Guntur	2013	Asia-I	261	1.37 ± 0.13	2.78 (4)	130 (95–183)	11.81 (6.56–22.73)

^a^Chi-square test for linearity of the dose–mortality response: ***P < 0.001, ** P < 0.01, *P < 0.05.

^b^Degrees of freedom.

^c^Resistance ratios (RR) with 95% confidence limits indicating the fold-difference for each insecticide in comparison to the most susceptible population at LC_50_ (RR = Asia-I populations of 2013 or 2012 divided by most susceptible Asia-I population in 2010). Confidence limits that include 1.0 indicate no significant difference from the susceptible population (Lethal ratio test-Robertson *et al*.^85^).

**Table 7:** Log-dose (mg L^–1^) probit model fitted to mortality data of Sriganganagar *B. tabaci* populations collected during 2010 to 2013.

Population	Sampling year	Genetic group	N	Slope ± SE	χ^2 ^a (df)^b^	LC_50_ (CI 95%)	RR^c^
**Cypermethrin**							
Sriganganagar	2010	Asia II-1	266	3.17 ± 0.55	1.11 (5)	472 (351–600)	1.00
Sriganganagar	2012	Asia II-1	288	1.65 ± 0.21	5.59 (5)	998 (666–1649)	2.12 (1.42–3.17)
Sriganganagar	2013	Asia II-1	290	1.32 ± 0.12	8.20 (5)	1362 (741–3734)	3 (1.83–4.55)
**Triazophos**							
Sriganganagar	2010	Asia II-1	266	1.53 ± 0.17	7.63 (5)	190 (109–363)	1.00
Sriganganagar	2012	Asia II-1	260	2.56 ± 0.40	1.61 (5)	394 (292–511)	2.08 (1.33–3.25)
Sriganganagar	2013	Asia II-1	336	2.53 ± 0.34	3.43 (4)	525 (402–647)	2.77 (1.81–4.24)
**Imidacloprid**							
Sriganganagar	2010	Asia II-1	274	1.76 ± 0.19	4.35 (5)	263 (100–592)	1.00
Sriganganagar	2012	Asia II-1	291	1.46 ± 0.17	2.33 (4)	513 (352–741)	1.95 (1.22–3.10)
Sriganganagar	2013	Asia II-1	221	2.11 ± 0.20	3.14 (5)	901 (581–1958)	3.42 (1.77–6.47)

^a^Chi-square test for linearity of the dose–mortality response: ***P < 0.001, **P < 0.01, *P < 0.05.

^b^Degrees of freedom.

^c^Resistance ratios (RR) with 95% confidence limits indicating the fold-difference for each insecticide in comparison to the most susceptible population at LC_50_ (RR = Asia-II-1 populations of 2013 or 2012 divided by most susceptible Asia-II-1 population in 2010). Confidence limits that include 1.0 indicate no significant difference from the susceptible population (Lethal ratio test-Robertson *et al*.^85^).

**Table 8:** Log-dose (mg L^–1^) probit model fitted to mortality data of New Delhi *B. tabaci* populations collected during 2010 to 2013.

Population	Sampling year	Genetic group	N	Slope ± SE	χ^2 ^a (df)^b^	LC_50_ (CI 95%)	RR^c^
**Cypermethrin**							
New Delhi	2010	Asia II-1	205	2.34 ± 0.27	4.10 (4)	31 (21–46)	1.00
New Delhi	2012	Asia II-1	245	2.14 ± 0.29	3.39 (4)	157 (113–210)	5.10 (3.42–7.59)
New Delhi	2013	Asia II-1	290	1.51 ± 0.12	12.82* (5)	194 (94–350)	6.30 (4.08–9.65)
**Triazophos**							
New Delhi	2010	Asia II-1	337	1.49 ± 0.16	9.83 (5)	124 (63–243)	1.00
New Delhi	2012	Asia II-1	245	1.32 ± 0.23	3.86 (4)	285 (183–449)	2.30 (1.33–4.00)
New Delhi	2013	Asia II-1	228	2.22 ± 0.37	5.57 (5)	324 (199–517)	2.62 (1.64–4.18)
**Imidacloprid**							
New Delhi	2010	Asia II-1	230	1.47 ± 0.17	7.62 (4)	55 (24–11)	1.00
New Delhi	2012	Asia II-1	347	1.24 ± 0.17	4.46 (4)	234 (135–477)	4.30 (2.53–7.31)
New Delhi	2013	Asia II-1	247	1.52 ± 0.22	3.64 (5)	178 (122–296)	3.20 (1.76–5.24)

^a^Chi-square test for linearity of the dose–mortality response: ***P < 0.001, **P < 0.01, *P < 0.05.

^b^Degrees of freedom.

^c^Resistance ratios (RR) with 95% confidence limits indicating the fold-difference for each insecticide in comparison to the most susceptible population at LC_50_ (RR = Asia-II-1 populations of 2013 or 2012 divided by most susceptible Asia-II-1 population in 2010). Confidence limits that include 1.0 indicate no significant difference from the susceptible population (Lethal ratio test-Robertson *et al*.^85^).
